# Supplementary figures and images for: Effect of experimental, morphological and mechanical factors on the murine spinal cord subjected to transverse contusion: A finite element study
Source: PLoS One. 2020 May 11;15(5):e0232975. doi: 10.1371/journal.pone.0232975 (PMC7213721; doi:10.1371/journal.pone.0232975)

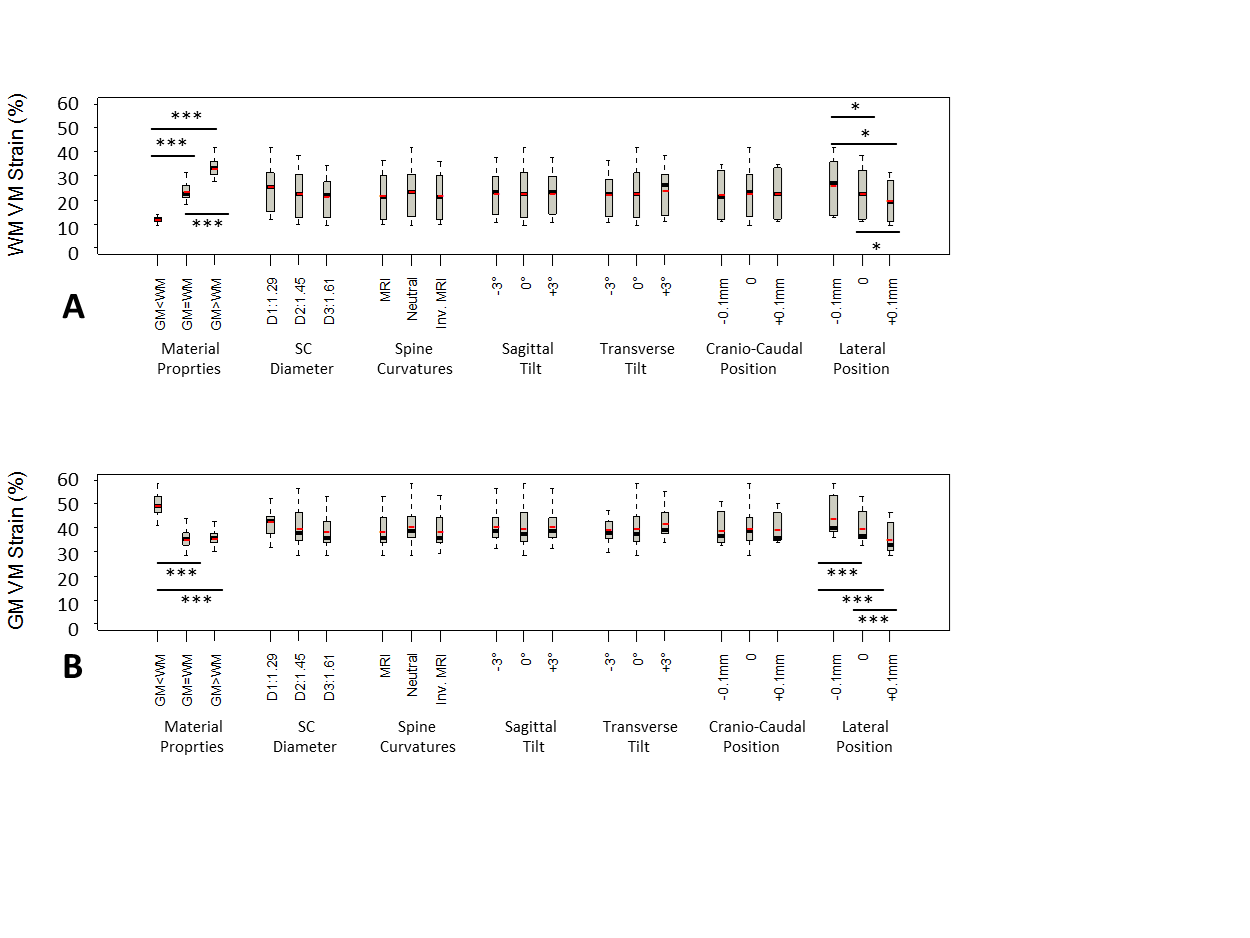

Supplement: S1 Fig — Values were obtained from WM ROIs(A) and GM ROIs(B). p-value was assessed through Wilcoxon test. ***: p<0.001, **:p<0.01, *:p<0.5. (TIF) [file pone.0232975.s001.tif]

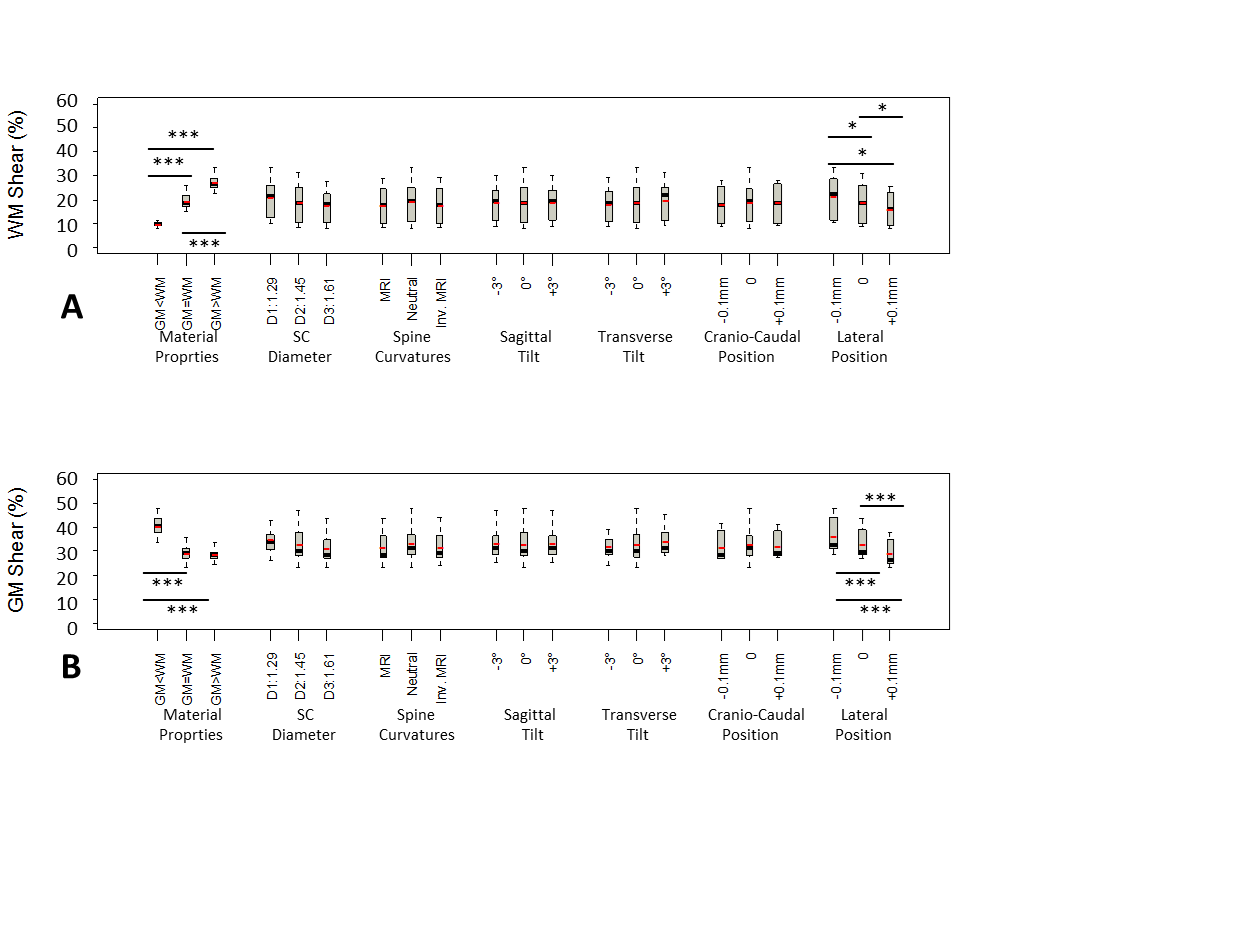

Supplement: S2 Fig — Values were obtained from WM ROIs (A) and GM ROIs (B). p-value was assessed through Wilcoxon test. ***: p<0.001, **:p<0.01, *:p<0.5. (TIF) [file pone.0232975.s002.tif]
