# Supplementary material for: Effect of experimental, morphological and mechanical factors on the murine spinal cord subjected to transverse contusion: A finite element study
Source: PLoS One. 2020 May 11;15(5):e0232975. doi: 10.1371/journal.pone.0232975 (PMC7213721; doi:10.1371/journal.pone.0232975)
Supplement: Attachment — Submitted filename: Response_to_Reviewers.docx [file pone.0232975.s003.docx]

Dear Academic Editor and Reviewers,

Thank you for the consideration of our manuscript. We appreciate your inputs which will help the manuscript to reach a wider readership. We would like to thank the reviewers for their careful review of the manuscript. Each point was carefully taken into consideration and answered in this letter and the revised version of the manuscript. Changes in the manuscript are highlighted in red.

Response to the Academic Editor :

- This is a rather technical report and an overview of the highlights is recommended for the broader readership, whether in abstract or introduction/discussion.

Lines 16-19 and 27-30 of the abstract were revised so that both the methodologies and the conclusions are more straightforward and accessible for a broader readership.

Opening of the discussion (lines 247-257) was also revised to better highlight the results and their meanings.

- Notably, the sentence "factors on the spinal cord behavior subjected to transverse contusion" seems inappropriate when no behavior of the animals is assessed in this report.

Line 20 and line 144: The term « behavior » was replaced with «mechanical behavior » " to ensure that there can be no ambiguity on the field of study.

Response to Reviewer #1

- While the authors do utilize previous work, it would be helpful to provide more details (sex, age, actual experimental results, etc.) on these prior experiments so as not to require the reader to search for these studies.

Lines 163-165: More information on the experimental model was provided in the manuscript: sex and age of the animals, as well as details regarding the surgical procedure and the experimental setup.

Lines 170: A sentence was added to inform the reader that all experimental data are provided in Fig2.

- The author should provide some further discussion on potential outcomes if injury magnitude and location were to change. How would this impact the model? Is the generated model only good for lateralized cervical contusion injuries?

Line 322-326 : Potential outcomes were provided in the “Limitations” part of the Discussion. The model was validated within a range of loading conditions and therefore could be used under different loading conditions as long as they fall within this range. In particular, simulation of non-lateralized contusion could be carried out as this loading mode is the same as the one validated. However, simulation of a contusion with a compression higher than 50% would require further validation.

Response to Reviewer #2

- It is interesting to note that the model falls within experimental results regardless of whether the assumptions of gray vs. white matter strain properties are reversed (gray> white and gray >>> than white, does the model still fall within experimental results.

Indeed, the three chosen sets of material properties were chosen to cover plausible range and ratios of WM and GM mechanical behaviors reported in the literature. Exacerbating the ratio between gray and white matters (for example increasing significantly gray matter stiffness as compared to white matter as suggested in your comment) may obviously increase the global stiffness of the cord and potentially fall outside of the experimental corridor. What we know is that it will strongly affect the strain and stress distribution within the cord. This actually, as stated in the discussion (line 263-266), would further demonstrate the strong impact of the choice of material properties and the need for further experimental investigations to better described the WM and GM properties. A sentence was added in the Discussion (lines 259-261), highlighting your observation that it is possible to simulate the global mechanical behavior of the spinal cord regardless of the gray vs white matter ratios tested in this study.
